# The effects of *Meloidogyne incognita* and *Heterodera glycines* on the yield and quality of edamame (*Glycine max* l.) in Arkansas

**DOI:** 10.21307/jofnem-2020-012

**Published:** 2020-03-17

**Authors:** J. E. Wilkes, T. L. Kirkpatrick

**Affiliations:** 1 105 Collins St., 200 Biosystems Research Complex, Clemson University, Clemson, SC 29634; 2 University of Arkansas, Southwest Research and Extension Center, 362 Highway 174 North, Hope, AR 71801

**Keywords:** Detection, Diagnosis, Food quality, *Glycine max*, *Heterodera glycines*, *Meloidogyne incognita*, Root-knot nematode, Soybean cyst nematode, Vegetable soybean

## Abstract

In 2012, the first domestic commercial edamame processing plant was established in Arkansas and edamame production was contracted out to local growers. Although the state is a major soybean producer, studies of nematode effects on edamame are limited. A survey of nematode genera and density in 64 contracted edamame production fields was conducted in 2013 and 2014. In both years, *Meloidogyne* and *Heterodera* were present in less than half of the surveyed fields while *Pratylenchus* was the most prevalent in 2013 and *Helicotylenchus* in 2014. A microplot study was conducted in 2014 in two locations to evaluate the effects of root-knot nematode (*Meloidogyne incognita, race 3*) and soybean cyst nematode (*Heterodera glycines*, HG type 2.5.7) on plant growth, yield and food quality components of edamame. Yield was the most consistent factor influenced by nematode pressure with increasing nematode population densities resulting in suppressed pod and seed weight. Additionally, seed protein content was reduced in the highest tested population density of *H. glycines*. In greenhouse studies, 22 advanced edamame breeding lines from the University of Arkansas soybean breeding program were compared with two susceptible commercial cultivars for suitability as hosts for both *M. incognita* and *H. glycines* independently. Four lines showed consistent reductions in *M. incognita* reproduction relative to the commercial cultivars and could represent sources of moderate resistance for development of future root-knot nematode resistant edamame cultivars.

Edamame, also known as vegetable soybean, is a high-fiber, low-sugar snack that has gained popularity in the USA in recent years. Not only does edamame have a high protein content but it also contains all the essential amino acids the human body requires for proper nutrition (Dixit et al., 2011). Compared to traditional soybeans grown for oil or grain production that are harvested at reproductive maturity, edamame is a shorter-season crop that is harvested before the seed is mature. Edamame is harvested when the pods at the upper nodes are filled with green seeds, before the pods start to turn yellow and senesce. Because edamame is most commonly marketed in the pod, the condition and quality of both the pods and seeds is of major importance. Although edamame has been grown in the USA since the late 1800s (Conlon, 2009), its popularity has recently increased because it is a high protein, vegetable-based, healthy food. From 2003 to 2007, US gross sales of edamame increased from $18m to $30m (Bernick, 2009), with the majority of the product purchased frozen, either in-pod or shelled.

Soybean protein is comparable to animal-sourced protein and contains all the amino acids necessary for a complete human diet with less cholesterol and saturated fats than animal protein (Dixit *et al.*, 2011). On average, a dry traditional soybean contains 35 to 40% protein. By contrast, edamame contains 33 to 36% protein on a dry weight basis (Rao *et al.*, 2002). Edamame has about 56% more protein and higher levels of calcium and iron than green peas (Masuda, 1991). The lipid content in vegetable soybean ranges from 13 to 16% of the dry weight (Rao et al., 2002). In addition to protein and oil content, a number of other health-related parameters are linked to quality edamame such as isoflavone, fiber and vitamin E (Johnson et al., 1999; Young et al., 2000). Important edamame appearance parameters include large beans, bean color, pod pubescence color and average number of beans per pod (Johnson et al., 1999). Flavor characteristics include components of sweetness, aftertaste, nuttiness and oiliness (Young et al., 2000).

Because edamame is harvested in the pods prior to complete seed maturation, pest management concerns and strategies are different from those routinely used in traditional soybean production systems. Plant-parasitic nematodes are a significant pest concern to the profitability of soybean production in the state of Arkansas (Kirkpatrick et al., 2014), and in the southern USA (Wrather and Koenning, 2003, 2006). Two nematodes of major concern for soybean production in Arkansas include the soybean cyst nematode (*Heterodera glycines*, Ichinoe) and the southern root-knot nematode (*Meloidogyne incognita*, Kofoid & White, Chitwood). Both nematodes have been historically present in Arkansas soybean and cotton fields (Kirkpatrick et al., 1992; Bateman et al., 2000; Walter and Barker, 1994; Tylka and Marett, 2014). A survey from 2018 identified that soybean cyst and root-knot nematode combined attributed to an average of 2.5% out of the 7.77% of total soybean yield loss due to disease in the Southern USA (Allen et al., 2018). From 2003 to 2005, the number one leading pathogen in soybean grown in the southern USA was the soybean cyst nematode (Koenning and Wrather, 2010). Currently, there is effort to actively monitor and manage soybean cyst nematode populations across the USA with The SCN Coalition^TM^, particularly as resistant varieties of soybean begin to lose their efficacy (Yan and Baidoo, 2018).

Although there have been studies on quality components of edamame (Li et al., 2012; Zeipina et al., 2017), the effect of nematodes on these quality traits has not be examined. A direct and widely used method of observing effects of nematode infection on a host is by identifying the nematode reproduction index (rate of initial to final nematode population densities (RI = Pi/Pf)) and relate the RI to the impact of plant yield. A similar approach can be taken to observe effects of RI on the quality of a plant host. Model systems have been used to describe the impact of nematodes on plant growth and yield (Seinhorst, 1988; Van de Berg, 2012). One of the most useful aspects of a disease-pressure model system is the estimation of a point at which the yield is threatened, also known as the damage threshold. Understanding the damage threshold of a specific host and cultivar allows growers or crop advisors to assess properly the nematode-associated risks and aid in determining when nematode management is needed. Many factors, in addition to nematode population density, play a role in determining the threshold including soil type, temperature, time, and host (McSorely and Duncan, 1995).

The goals of this study were to document the nematodes that were present in Arkansas edamame fields and determine the effect of two major nematode species on edamame yield and quality. The objectives were to: survey edamame production areas in Arkansas for plant-parasitic nematodes, determine the effects of root-knot and soybean cyst nematode initial population densities on the yield and food quality of edamame in microplots and evaluate edamame breeding lines from the University of Arkansas soybean breeding program for host suitability to *M. incognita*, host race 3, and *H. glycines*, race 5 (HG type 2.5.7), independently.

## Materials and methods

### Survey of nematodes in edamame production fields

Commercial edamame fields under contract with the American Vegetable Soybean and Edamame, Inc. (33 fields in 2013 and 31 fields in 2014) were surveyed for the presence and density of plant-parasitic nematodes. All sites were sampled within three weeks after edamame harvest. Samples were collected using a 2.5-cm-diameter soil probe (Soil Sampler, Step Made, USA) to a depth of 15 to 20 cm. Six cores were collected for each individual sample along the rows where the soybean stalks remained using a zig-zag pattern (Van Bezooijen, 2006) with one site representing 4 to 8 hectares, averaging a total of 500−600 cm^3^ of soil. Samples were placed in plastic bags, labeled with field name and location within field, transported to the laboratory in an insulated ice chest and stored in a cooler at 10−13°C (Barker et al., 1969). Soil samples were processed within two weeks of collection date. For each sample, a 100-cm^3^ aliquot from the mixed composite sample was assayed using a semi-automatic elutriator (Byrd et al., 1976) equipped with 40-mesh (0.4 mm) sieve over a 400-mesh (0.037 mm) final collection sieve. Soil collected on the 400-mesh sieve was processed using centrifugation flotation (Jenkins, 1964) and nematodes were identified to genus using a dissecting microscope at 60−80×.

### Impact of *H. glycines* and *M. incognita* on edamame yield and quality in microplots

Field microplot experiments were conducted at two locations in 2014 to examine the effect of *M. incognita* and *H. glycines* on edamame yield and food quality. Each location included independent microplot experiments of *M. incognita* and *H. glycines*. Both experiments were arranged in a complete randomized block design with six replications of four levels of initial population densities.

Location 1 was at the University of Arkansas Southwest Research and Extension Center near Hope, AR. Microplots were made from square clay flue tiles (37.5 cm×37.5 cm) buried 122 cm deep in a grid pattern of 5×10 and spaced 91 cm apart. Each microplot was filled with a mixture of silt loam topsoil (48% sand, 50% silt, 2% clay) mixed 50:50 (v/v) with coarse sand to a volume of 21,093.75 cm^3^. Prior to filling the plots, the soil mix was steam pasteurized for 30 min at 70°C. The entire block was enclosed in metal fencing to deter pests. During the first month after seeding, the block was covered with nylon netting to deter birds. Location 2 of this study was a previously established plot on the Arkansas Agricultural Research and Extension Center in Fayetteville, AR. At this site, the microplots were of the same size and material as in Location 1. Existing soil from each plot was removed with a shovel and the inside surface of each tile was drenched with a 10% chlorine bleach (NaOCl) solution two months prior to refilling and planting. The microplots were filled with soil (86% sand, 11% silt, 3% clay, bulk density of 1.2 g/cm^3^) obtained from the Arkansas River Valley near Van Buren, AR, that had been pasteurized as described above. The site was fenced to deter pests.

Inoculum of *M. incognita* for both locations was prepared by collecting nematode eggs and second-stage juveniles from 60-day-old greenhouse stock cultures on tomato (*Solanum lycopersicum* L. cv “Rutgers”). The culture was originally collected in 2006 on cotton in Desha County, AR. Inoculum for the microplots was prepared as follows: tomato plants growing in 60-cm-diameter clay pots were removed and the above-ground vegetation discarded. Galled roots were separated from the soil and cut into 2 to 3 cm segments and mixed back into the soil from the pots, creating a soil inoculate densely populated with *M. incognita*. The population density of the soil inoculate was determined by collecting 100 cm^3^ core samples and using a semi-automatic elutriator to collect root pieces and second-stage juveniles. Root pieces collected on the 40-mesh sieve were processed using NaOCl (Hussey and Barker, 1973) to quantify eggs. This process of sampling was repeated three times, mixing in between sampling, to estimate nematode population density of the prepared soil inoculate. Nematode treatments consisting of varying initial population density (Pi) were: 0 (control), 1,000, 10,000 and 100,000 *M. incognita* eggs and juveniles per microplot or 0, 70, 700 and 7,000 infective units per 100-cm^3^ soil in each microplot. The amount of soil inoculate needed for treatment of each plot was measured and bagged, adding pasteurized soil to the lower treatments to reach a uniform volume of 704 cm^3^ for each treatment. Treatments were applied to each plot by incorporating the measured soil inoculate into the upper 10 cm of soil in each microplot with a shovel and rake.

Inoculum for *H. glycines* (Race 5, HG type 2.5.7) for both locations was obtained from a stock culture maintained by Dr R.T. Robbins, University of Arkansas. Cysts were extracted from susceptible soybeans grown for 40 days after inoculation in a greenhouse and stored in moist sand at 4°C. Race 5 (HG type 2.5.7) was chosen for this experiment since it has been recorded frequently in Arkansas fields (Bateman and Kirkpatrick, 2010). Cysts were extracted by mixing the soil in a bucket of water and pouring the suspension through 30-mesh (0.595 mm) over 60-mesh (0.25 mm) nested sieves. The number of eggs per cyst was estimated by crushing a known number of cysts and counting the eggs released. Based on the average egg count per cyst, the number of cysts needed for each plot treatment was calculated. Cysts were placed into a 500-mL bottle with 100 mL of tap water and poured into the assigned microplots. An additional 500 mL of clean tap water was used to rinse out the bottle and poured into the respective microplot. The added cysts were incorporated into the upper 10 cm of soil in each microplot using a shovel and rake as described above for the root-knot inoculation. Initial population densities were 0, 1000, 10,000 and 100,000 eggs per microplot as with the *M. incognita* treatments.

Immediately after inoculation, six seeds of the edamame cultivar “8080”, which was the most common cultivar grown by Arkansas growers, were planted in each plot about 7 cm apart. At 18 days after seedling emergence, the plant population was adjusted to four plants per plot. Plots were treated with a mild insecticidal soap to discourage an aphid infestation (“Bug B Gon”, Ortho®) two weeks after seedling emergence. A complete fertilizer (Scott’s Osmocote® 14-14-14) was applied as directed in label one week after seedling emergence as suggested by soil test results. All plots were hand-watered as necessary throughout the study.

#### Nematode sampling and evaluation

The nematode population density from all plots at both locations was assayed four weeks after planting to monitor populations halfway through the season. Soil samples from each microplot were obtained by collecting six individual cores using a soil probe (2.5-cm-diameter) 15 cm deep. To avoid contamination between plots, the soil probe was washed and sterilized with 10% chlorine bleach after each plot was sampled. The risk of significant contamination was further minimized by first taking the samples from the nematode-free control plots and then sampling each treatment separately from low to high population density. The cores were bulked for each plot and a 100-cm^3^ subsample was assayed using a semi-automatic elutriator followed by centrifugal flotation. Juvenile and egg counts were recorded for each plot. Soil was sampled from each plot again on the same day as harvest before the plants were removed from the plots to quantify nematode densities at time of harvest. Cross contamination was prevented in the same manner as described above. The roots from *M. incognita*-infested plots were scored for galling severity using a 0–10 severity scale (Barker, 1982), and the roots were placed in a paper bag, and transported to the laboratory. Nematode eggs were extracted from roots from each plot using NaOCl as described previously. The average number of eggs per root system in each plot was recorded (Table 2).

#### Microplot harvest

Soybeans in the microplots were harvested by hand-picking all pods from each plant at soybean growth stage R6 (Fehr, 1971) that occurred 73 days after planting (Location 1) and 75 days after planting (Location 2). The number of pods per plant was recorded, and all pods from each plot were bulked, placed into a paper bag and labeled. The number of pods containing one, two and three seed and total weight of all pods were recorded per plot. All pods were hand-shelled, and the beans were bulked for each plot. The total fresh seed weight was recorded.

The fresh seeds from all pods were arranged in foil boats by plot and put in a blast freezer (Air-O-Chill, Elextrolux Professional SpA, Pordenone, Italy) at −25°C for 20 min. The seeds were then placed in a freeze dryer (Virtis Genesis, SP Scientific, PA, USA) at −45°C for a total of 120 hr (Rayaprolu et al., 2015). The moisture content was calculated as the weight difference before and after freeze drying. The freeze-dried seeds were ground using an electric coffee bean grinder for approximately 30 sec and sieved through a 30-mesh (0.595 mm) sieve. The sieved flour was used for analysis of protein (AOAC 990.03), lipid (AACC 30-26) and starch (Megazyme kit, AACC 76-13.01, 1995) as follows: the total amount of protein in each sample was measured using the nitrogen combustion methodology (CLG-PRO4.03) (AOAC method 992.23, AOAC, 1990). The lipid extraction protocol was modified from the AACC method, “Crude Fat in Soy Flours” (AACC method 30-26, AACC, 1995). Filter paper (Whatman^TM^ 4, 150 mm, Cat No 1004 150) was folded, weighed, and a 2-gram sample of the flour was wrapped in the filter paper. The wrapped sampled was inserted into a paper tube and placed in a soxhlet apparatus placed under an enclosed vent hood. The system included condensers, filter tubes and 500-mL glass collection beakers on a series of hot plates (AACC method 30-26). Approximately 150 mL of petroleum ether was added to each sample via the condensers, and the samples were run through a 5-hour cycle to ensure removal of all soluble lipids. The samples wrapped in filter paper were then aired dried for a minimum of 8 hr and weighed. The amount of lipid in each sample was calculated by the change in sample flour weight to four decimal places. The starch content was analyzed using a Megazyme©, 2011 Total Starch kit (amyloglucosidase/*α*-amylase method, Megazyme, Bray Business Park, Bray, Co. Wicklow, A98 YV29, Ireland) to quantify the total starch in each sample as directed, corresponding to the AACC Method 76-13.01. Light absorption from each sample was measured using a spectrophotometer (Shimadzu UV160 UV-Vis, Dual Beam) at a wavelength of 500 nm.

All collected data were analyzed using general linear models (SAS Institute, Inc., 2014). Differences among treatments were compared using least significant difference (LSD) at *p* ≤ 0.05.

### Host suitability of edamame breeding lines

In total, 24 advanced breeding lines of edamame from the University of Arkansas, Division of Agriculture soybean breeding program were obtained from Dr Pengyin Chen, Department of Crops, Soils, and Environmental Sciences, University of Arkansas, Fayetteville, AR. Three seeds of each line were planted in 10-cm-diameter clay pots filled with pasteurized sandy loam soil (86% sand, 11% silt, 3% clay, bulk density of 1.2 g/cm^3^). One week after planting, plant populations were adjusted to one plant per pot. In all trials, the pots were arranged in a randomized complete block design to account for possible variability in environmental conditions at different positions on the greenhouse bench. Five replications of each line were infested with nematodes as described below. Plants were watered and fertilized with Scott’s Osmocote® 14-14-14 (Scott’s MiracleGro^TM^) as needed for active growth. Greenhouse temperatures ranged from 26–29 °C for the trials conducted in June–July, 2013, and 24–27°C for the trial repeated in October–December, 2013. All lines were evaluated in a third trial May–June, 2014.

#### Meloidogyne incognita

A population of *M. incognita* that was originally collected from soybean in Drew County, AR, and maintained on tomato, cv “Rutgers” was used for evaluation of suitability of the lines as hosts for root-knot nematodes. Eggs were collected from 60-day-old, greenhouse-grown plants using the following methodology: galled roots were removed from pots, rinsed thoroughly to remove soil, placed in a blender with 100-cm^3^ water, and pulsed for three, 1-second cycles. The root pieces were then placed in an Erlenmeyer flask and extracted by shaking in a 0.5% solution of NaOCl for 2.5 min to free eggs from egg masses (Hussey and Barker, 1973). The eggs were collected on a 500-mesh (0.025 mm) sieve and rinsed thoroughly with tap water. Each pot was inoculated by pipetting 4,500 eggs in 5 mL water in two 1-cm diameter holes near the base of the plant at true leaf stage. Plants were grown in a greenhouse for the duration of the experiment. The edamame cultivar, “8080”, was the susceptible check used in all trials.

After 45 days, the plants were cut at the soil line and the stem and root tissue was weighed for fresh weight. The roots were then rated for root galling severity on a 0–10 scale where 0=no galls, and 10=100% of the roots covered with galls (Barker, 1982). Following gall ratings, roots were placed whole into a 500-mL wide-mouth Erlenmeyer beaker and stirred with an electric mixer in 0.5% NaOCl for 4 min. The solution and roots were poured through a 100-mesh (0.149 mm) over 500-mesh (0.025 mm) stacked sieves and rinsed with tap water. The eggs collected on the 500-mesh sieve were quantified at 60× magnification. The roots were then dried for 48 hr at 60°C in a plant drier and weighed. Root weights were used to calculate the nematode reproduction per gram of dry root tissue.

#### Heterodera glycines

The same stock culture of *H. glycines* (HG type 2.5.7) that was used in the microplot studies was also used for the host suitability trial. Cysts that had previously been extracted from greenhouse-grown soybean plants and stored in 4°C in sterile soil were obtained from Dr R.T. Robbins (University of Arkansas, Fayetteville, AR). Cysts were separated from the soil by rinsing on stacked 30-mesh (0.595 mm) over 60-mesh (0.25 mm) sieves. The cysts collected on the 60-mesh sieve were transferred into a glass tissue grinder and lightly ground to free the eggs, and the eggs were collected on a 500-mesh (0.025 mm) sieve. The inoculation procedure was performed in a manner identical to the root-knot procedure above at a rate of 4,500 eggs per pot.

After 30 days, plants were cut at the soil line and each pot was extracted separately by placing the roots and soil into a 4.7-liter bucket. Water was added at high pressure, and the soil and roots were mixed vigorously and poured through 30 over 60-mesh nested sieves with each sieve rinsed thoroughly (Hussey et al., 1991). Cysts were collected from the 60-mesh sieve in 100-mL beakers and stored at 4°C until quantified. The contents of the beaker were poured into a counting dish, and cysts were counted. After extraction, the roots of each plant were dried for 48hr in a large plant drier at 60°C and weighed.

The five replications within each trial were analyzed in an ANOVA using JMP ® 14.1.0 (SAS Institute Inc., 2018) for both root-knot and soybean cyst nematode trials, independently.

Data for the three root-not trials were recorded as gall rating and average egg count per gram of root tissue across all five replicates. For the cyst trials, the average number of cysts collected from the five replicates was calculated for each trial and an ANOVA was used for each trial independently. Lines that performed significantly different within each trial were identified using LSD analysis (*α*=0.05).

## Results

### Survey of plant-parasitic nematodes in Arkansas edamame production fields

A total of 203 soil samples were collected and processed from edamame fields in two years. The nematode genera found in both years of the survey were *Meloidogyne*, *Heterodera*, *Pratylenchus*, *Helicotylenchus*, *Tylenchorhynchus*, *Paratrichodorus* and *Hoplolaimus* (Table 1). Other plant-parasitic nematodes identified in low levels in only one or two fields include *Xiphinema* and *Criconemoides* (data not shown). *Meloidogyne* and *Heterodera*, both of which are economically significant nematodes in traditional soybean production systems, were found in edamame fields both years of the survey. In 2013, *Meloidogyne* was found in 12 (of 33) fields and *Heterodera* was found in 13 fields. In 2014, *Meloidogyne* was present in fewer fields (7 fields out of 31 sampled) and *Heterodera* was found in 12 fields. *Meloidogyne* was found in the highest abundance in individual fields in both 2013 and 2014 in Lonoke, Johnson, and Yell counties (Table 1). *Meloidogyne* was not detected in Faulkner, Pulaski or Logan counties in 2013, nor in Logan and White counties in 2014. *Heterodera* was common both years although they were not detected in Faulkner and Yell County in 2013 nor in Logan County in 2014. *Heterodera* was found in highest numbers in Lonoke County both years. Other less economically important parasitic species, such as *Helicotylenchus* and *Pratylenchus* were detected in more fields (higher prevalence) for both years.

**Table 1.** Average population density of nematode genera (when present) identified in Arkansas edamame fields.

County	No. of fields	*Meloidogyne*	*Heterodera*	*Pratylenchus*	*Helicotylenchus*	*Tylenchorhynchus*	*Paratrichodorus*	*Hoplolaimus*
*2013*								
Faulkner	1	0 a	0	94	42	83	42	0
Johnson	1	42	83	83	42	0	0	0
Logan	10	0	42	50	42	80	42	0
Lonoke	9	278	54	77	88	236	125	125
Pulaski	1	0	125	83	250	167	125	0
White	7	42	52	0	0	0	42	0
Yell	4	339	0	369	42	83	42	0
Percent prevalent^b^		36.36	39.39	57.58	39.39	30.3	21.21	3.03
*2014*								
Johnson	7	2731	38	51	142	50	38	0
Logan	3	0	0	38	102	128	0	0
Lonoke	7	637	346	66	109	99	38	0
White	8	0	61.2	38	67	66	154	77
Yell	6	38	58	53	84	38	0	0
Percent prevalent		22.58	38.71	48.39	80.65	58.06	19.35	3.23

Notes: ^a^Mean number of vermiform nematodes per 100 cm^3^ soil in each collected sample in the respective county; ^b^the percent of fields that contained the nematode genera across all sampled locations.

### Impact of *Meloidogyne incognita* and *Heterodera glycines* on edamame yield and quality in microplots

#### Yield parameters

##### Meloidogyne incognita:

**Figure 1:**
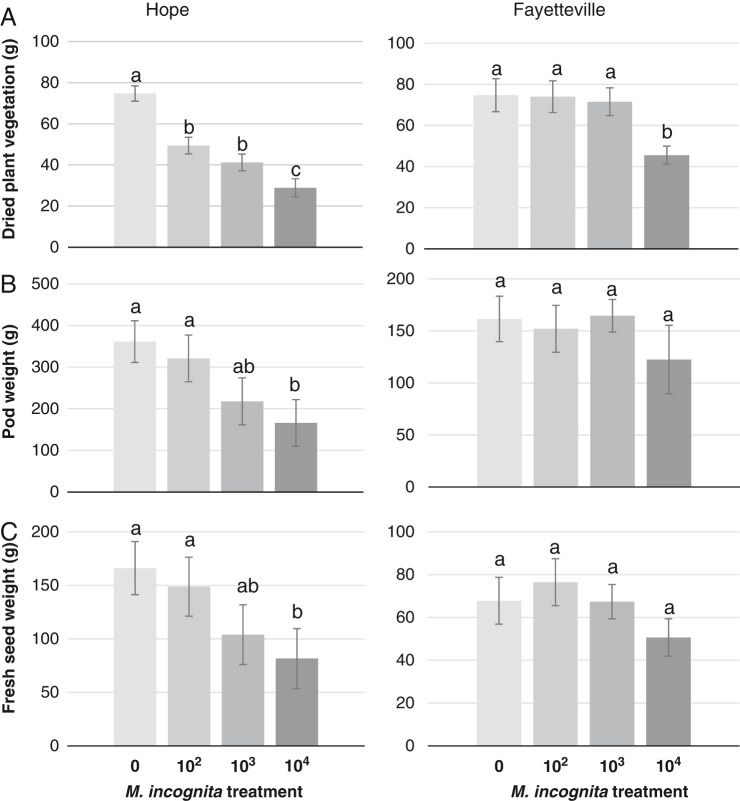
Effects of *Meloidogyne incognita* on three yield parameters; (A) dried plant weight in grams; (B) Total weight of harvested edamame pods in grams; and (C) Total fresh seed weight in grams on edamame cultivar, ‘8080’, in microplots from two locations. Plants were harvested at reproductive stage 6 (R6). Error bars represent standard error of the mean for each treatment. Differences in letters indicate significantly different values as determined by LSD at p ≤ 0.05.

Location affected many of the tested variables, therefore data were analyzed and reported by site independently. The mean second-stage juvenile (J2) population density at harvest ranged from 79 to 110/100 cm^3^ soil at Hope and 2 to 1,018 J2s/100 cm^3^ of soil at Fayetteville in all infested treatments (Table 2). Root galling severity was significantly greater at the two higher initial infestation rates at Hope, and at the highest infestation rate at Fayetteville, compared to the lower infestation levels. No galling was seen in the uninfested control plots. Host suitability, as indicated by egg production on roots, was high in all infested treatments at both locations with some variability in the Hope location. Plant top dry weight declined with increasing initial nematode infestation levels at the Hope location, while only the highest initial density resulted in lower plant weights at Fayetteville (Fig. 1A). Dry root weights were not significantly different between treatments (data not shown). Fresh pod weight and fresh weight of shelled seed were suppressed by the highest initial infestation level at the Hope location with no significant effect in the Fayetteville location (Fig. 1B,C). Other data including the number of pods per plant, the number of seeds per 30 grams of pods and the ratio of 1-bean, 2-bean and 3-bean pods were not affected by *M. incognita* pressure (data not shown).

**Table 2.** *Meloidogyne incognita* population density in microplots at edamame harvest in two locations.

Initial infestation density (eggs per microplot)	*M. incognita* juveniles (J2) per 100 cm^3^ soil	Gall rating^a^	Average number of eggs from one root ball
*Hope*			
0	0 ^a,b^	0 ^a^	0 ^a^
1,000	92 ^b^	6.2 ^b^	1,681,720 ^b^
10,000	79 ^b^	8.3 ^c^	1,510,267 ^b^
100,000	110 ^b^	9.1 ^c^	682,973 ^a,b^
*Fayetteville*			
0	0 ^a^	0 ^a^	0 ^a^
1,000	2 ^a^	5.2 ^b^	200,720 ^b^
10,000	297 ^b^	7.0 ^b^	859,840 ^b^
100,000	1,018 ^c^	9.7 ^c^	1,559,313 ^c^

Notes: ^a^Root gall rating scale from 1 to 10 where 0 = no galling and 10=100% root system with severe galling; ^b^Means within columns for each location with the same letter do not differ significantly (p ≤ 0.05) by LSD.

##### Heterodera glycines:

**Figure 2:**
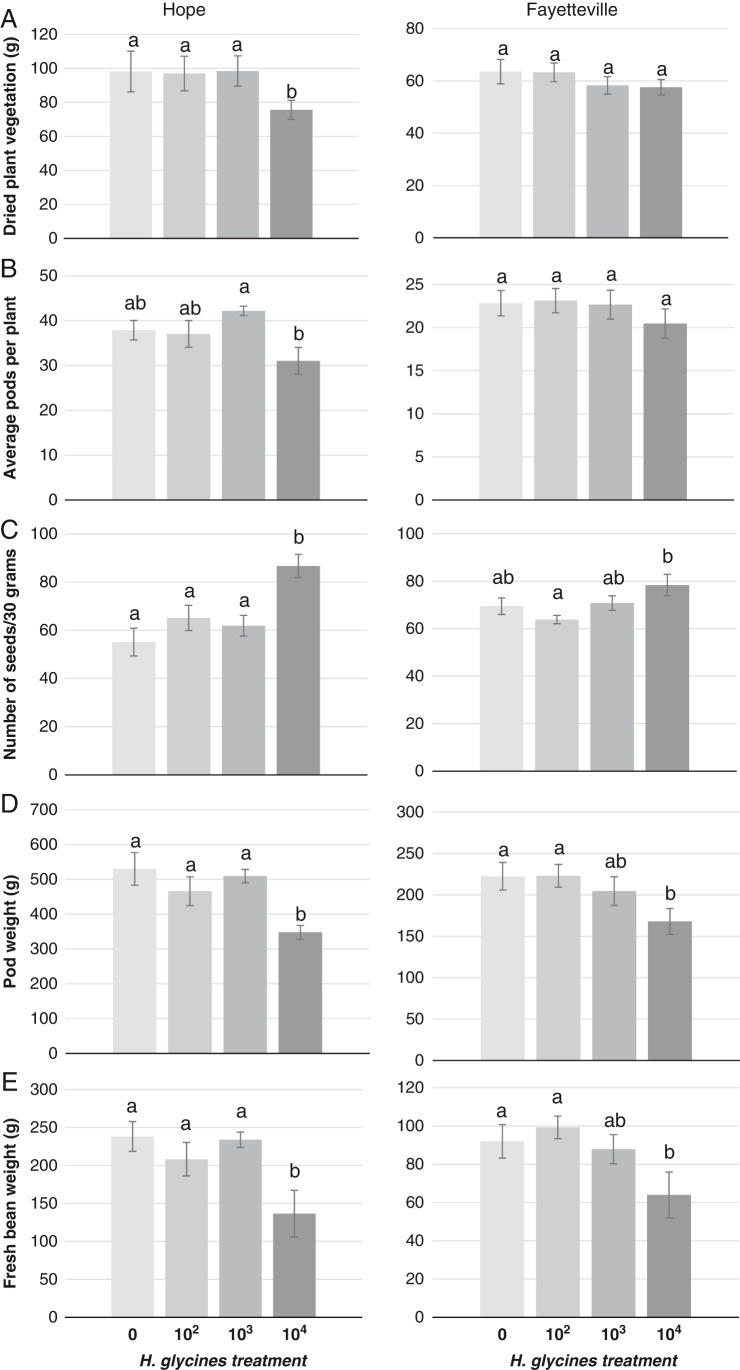
Effects of *Heterodera glycines* on five edamame yield parameters; (A) total weight of dried plant mass; (B) average number of harvested pods per plant; (C) the number of seeds in 30 grams; (D) total weight of pods in grams; and (E) total fresh bean weight in grams in microplots from two locations. Edamame plants were harvested at reproductive stage 6 (R6). Error bars represent standard error of the mean for each treatment. Differences in letters indicate significantly different values determined by LSD at p ≤ 0.05.

More J2s and cysts were recovered from both soil and roots in plots with the highest initial infestation levels at both locations (Table 3). The highest initial infestation level resulted in lower edamame plant top dry weights and lower average number of pods than for the all other treatments at the Hope location. There were no treatment effects on plant dry weight or pods at Fayetteville (Fig. 2A,B). The number of seeds per 30 grams of pods was greater at the highest initial infestation level at Hope, and higher than the intermediate infestation rate at Fayetteville (Fig. 2C). This measurement indicates that average seed size of the edamame was significantly smaller in higher nematode density treatments. Pod weight and fresh seed weight were suppressed at the highest initial infestation level compared to the control and lowest initial infestation level at both locations (Fig. 2D,E).

**Table 3.** *Heterodera glycines* population density in microplots at edamame harvest in two locations.

Initial infection level (eggs per microplot)	*H. glycines* juveniles (J2) per 100 cm^3^ soil	Cysts per 100 cm^3^ soil	Cysts from roots in microplots at harvest
*Hope*			
0	0 ^a^	0 ^a^	0 ^a^
1,000	2 ^a^	8 ^a^	97 ^a^
10,000	26 ^a^	67 ^a^	745 ^a^
100,000	199 ^b^	850 ^b^	7,674 ^b^
*Fayetteville*			
0	176 ^a^	14 ^a^	240 ^a^
1,000	102 ^a^	12 ^a^	220 ^a^
10,000	531 ^a^	36 ^a^	774 ^a^
100,000	2,068 ^b^	254 ^b^	3,923 ^b^

Note: ^a^Means within columns for each location with the same letter do not differ significantly (p ≤ 0.05) by LSD.

#### Edamame quality parameters

*Meloidogyne incognita* resulted in lower seed moisture and protein content at the two higher initial infestation rates at Hope, but not at Fayetteville (Table 4). *M. incognita* did not affect lipid or starch content at either location. In the *Heterodera glycines* trial, the seed moisture content was lower at the highest infestation level than at the lowest initial infestation level at both locations (Table 5). Seed protein content also decreased at both locations in the highest initial infestation density of soybean cyst nematodes compared to the control and lowest initial infestation level. Additionally, the seed lipid content was also lower in the highest initial *H. glycines* treatment at Fayetteville. There was no significant trend seen in starch levels in either location.

**Table 4.** Food composition percentage on a dry weight basis from edamame seed in *M. incognita*-infested microplots.

Treatment (eggs per plot)	Moisture	Protein	Lipid	Starch
*Hope*				
0	71.37 ^a^	38.24 ^a^	11.16 ^a^	10.29 ^a^
1,000	70.37 ^a^	38.23 ^a^	12.83 ^a^	10.56 ^a^
10,000	68.04 ^b^	36.89 ^b^	14.58 ^a^	10.08 ^a^
100,000	68.83 ^b^	36.46 ^b^	14.66 ^a^	10.06 ^a^
*Fayetteville*				
0	76.04 ^a^	36.53 ^a^	10.02 ^a^	9.27 ^a^
1,000	75.41 ^a^	36.44 ^a^	10.27 ^a^	9.56 ^a^
10,000	76.19 ^a^	36.92 ^a^	12.10 ^a^	9.43 ^a^
100,000	75.75 ^a^	36.47 ^a^	10.40 ^a^	9.58 ^a^

Note: ^a^Means within columns for a site differ significantly at p ≤ 0.05 by LSD.

**Table 5.** Food composition percentage on a dry weight basis from edamame seed in *H. glycines*-infested microplots.

Treatment (eggs per plot)	Moisture	Protein	Lipid	Starch
*Hope*				
0	70.15 ^a,b^	37.57 ^a^	10.14 ^a^	10.73 ^a^
1,000	70.61 ^a^	37.57 ^a^	10.85 ^a^	10.46 ^a^
10,000	69.59 ^a,b^	36.86 ^a,b^	10.38 ^a^	10.91 ^a^
100,000	66.24 ^b^	36.18 ^b^	11.66 ^a^	10.94 ^a^
*Fayetteville*				
0	74.87 ^a^	35.86 ^a^	8.03 ^a^	12.62 ^a^
1,000	75.33 ^a^	36.37 ^a^	7.82 ^a^	12.39 ^a^
10,000	74.82 ^a,b^	35.71 ^a^	8.87 ^a,b^	13.01 ^a^
100,000	74.46 ^b^	34.66 ^b^	9.62 ^b^	13.25 ^a^

Note: ^a^Means within columns for a site differ significantly at p ≤ 0.05 by LSD.

### Host suitability of edamame breeding lines

#### Meloidogyne incognita:

**Figure 3:**
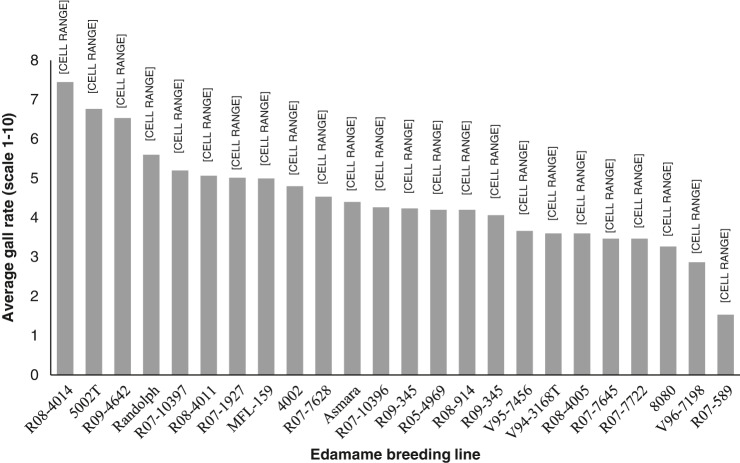
Average gall rating of *Meloidogyne incognita* on 22 different edamame breeding lines 45 days after inoculation. Gall ratings were based on a 1-10 scale (Barker, 1982). Each bar represents an average of three trials with five replications in each trial. Edamame lines with different letters listed above bars indicate significant differences using LSD analysis, p ≤ 0.05.

**Figure 4:**
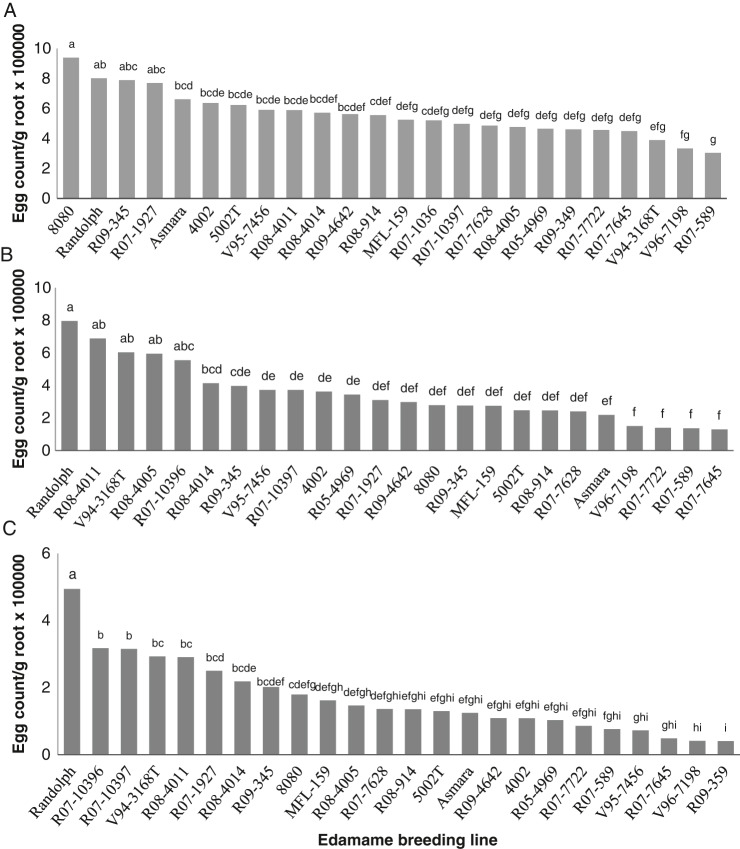
Average number of *Meloidogyne incognita* eggs per gram of dry root in 22 different edamame breeding lines 45 days after inoculation. Each bar in trial 1 (A), 2 (B), and 3 (C) represent an average of five replications. Edamame lines with different letters above the bar indicate significant difference as determined by LSD analysis, p ≤ 0.05.

The gall ratings from the three trials were not significantly different, therefore gall rating data are presented as the average. The maximum root gall rating over the three trials was 8.6 (“R08-4014”, in Trial 3), and the minimum gall rate was 1 (“R07-589”, in Trial 1). When comparing ratings across the three trials, the mean root galling rates were consistently lower in edamame lines “R07-589”, “V96-7198”, “R07-7645” and “R07-7722” compared to the other evaluated lines (Fig. 3), including the widely used commercial cultivar, “8080”. There was significant variation in the egg count per gram of root between the three root-knot trials (*p* < 0.0001), therefore each of the three trials were analyzed independently. In Trial 1, the maximum average egg count per gram of root tissue was 939,040 on line “8080” and the lowest egg count was from line “R07-589” with 304,400 eggs/gram of root. Based on paired *t*-tests, results from Trial 1 did not identify a line that had significantly lower reproduction compared to the other tested breeding lines (Fig. 4A). In Trial 2, the highest average egg count per gram of root tissue was 795,347 on line “Randolph” and the lowest egg count was on line “R07-7645” with 129,107 eggs/gram of root. As in Trial 1, analysis did not reveal any lines with significantly lower root-knot reproduction (Fig. 4B). In the final trial, the highest number of eggs per gram of root was 493,796 found on line “Randolph,” and the lowest nematode egg count was 40,449 from line “R09-359”. As in the first two trials, no significantly resistant line was identified, but “Randolph” was a significantly more suitable host than the other lines in the third trial (Fig. 4C). Numerically, the breeding lines “R07-589”, “V96-7198” and “R07-7645” were consistently low in root-knot reproduction.

#### Meloidogyne incognita:

**Figure 5:**
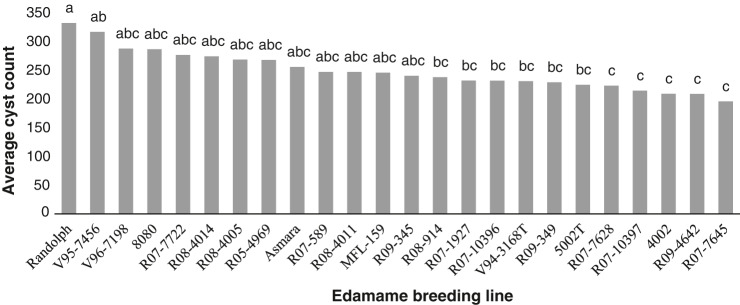
Average number of *Heterodera glycines* cysts from soil and roots of 22 edamame breeding lines 30 days after inoculation. Each bar represents the average of two trials with five replications in each trial. Different letters above bars indicate LSD significant levels, p ≤ 0.05.

There were no significant differences between the two repetitions, so the data were combined for analysis. The highest number of cysts per plant, 332, was collected from “Randolph” and the lowest cyst count of 195 was collected from “R07-7645”. All the evaluated soybean lines were suitable hosts for *H. glycines* (Fig. 5).

## Discussion

In the field survey, many nematode genera were found associated with edamame production. *Meloidogyne* and *Heterodera*, both known to be economically significant in traditional soybean production in the state, were found at levels that were well above the action threshold reported for Arkansas. While *Pratylenchus* were the most prevalent in all surveyed fields, reported damage on soybean has been inconsistent (Kirkpatrick et al., 2014). Further identification of the species of both *Pratylenchus* and *Meloidogyne* would offer more information on the potential damage on the soybean crop. Other nematode genera detected in the survey, including *Tylenchorhynchus*, *Heliocotylenchus* and *Paratrichodorus*, were also found frequently at relatively high densities, but these nematodes have not been reported to significantly impact yield of soybean. This survey helps demonstrate the importance of nematode sampling for growers to increase the awareness of parasitic nematode populations that exist in their fields before planting edamame and for developing management strategies to manage both root-knot and cyst nematode.

In the microplot study, *M. incognita* suppressed plant weight, number of pods and average seed weight per 30 g at the Hope location, but not at Fayetteville. It is likely that greater environmental stress, particularly high summer temperatures, may have affected the Hope location by exacerbating nematode effects. This same trend was apparent for *H. glycines* for plant weight and number of pods. Observed levels of *H. glycines* also may present a concern for reducing protein content in seed. Interestingly, there was an increase in lipid content where protein content decreased. This further supports our data as these two traits are known to be inversely related (Wilcox and Shibles, 2001). Additionally, it has been reported that infection with *H. glycines* reduces the formation of nitrogen-fixing nodules (Hussey and Barker, 1976), which may contribute to the reduction of protein levels in edamame beans. If growers are faced with soybean cyst in their fields and are using edamame cultivar “8080”, it is likely that heavy infection may result in lower yield and reduced protein content.

It was observed at edamame harvest that there was nematode contamination in the cyst nematode study at the Fayetteville location. Cyst nematodes were detected in the uninfested control treatment at levels not significantly different than the two lowest cyst-treated plots. The contamination of cyst is likely due to residual cysts from previous trials that were not eradicated in the decontamination prior to conducting the trial. This theory was confirmed by performing a host differential test using cysts collected from the uninoculated control plots and identifying the cysts to be of race 14 (H.G. type 1.3), as opposed to the Race 5 used in this experiment (data not shown). The data collected at Fayetteville did not have a successful control, and results may not be reliable. However, the repetition study in Hope does indicate there are possible effects of both root-knot and cyst nematode on edamame quality parameters.

In both soybean cyst and root-knot resistant assays, all lines were overall good hosts for *Meloidogyne incognita*, Race 3, and *Heterodera glycines*, HG type 2.5.7, based on their high nematode reproduction counts. However, relative to the tested germplasm, there were some trends that appeared consistent. Breeding line “Randolph” consistently performed as a very suitable host for both *M. incognita* and *H. glycines* with high nematode reproduction across all trials. Conversely, breeding lines “R07-589”, “V96-7198” and “R07-7645” were the least suitable hosts for *M. incognita*. Additionally, one breeding line, “R07-7645”, also showed low suitability for *H. glycines* reproduction.

“R07-589” produces a dark brown seed coat, a unique characteristic in vegetable soybean cultivars. The seed of edamame varieties that are currently on the market commercially in the USA are a vivid green, but there is no current market demand for dark-colored edamame. It is common for disease-resistant soybean lines to have the trait for a dark-colored bean (Shannon et al., 2004). Dark brown soybeans may contain a lower amount of protein but more oil per gram of seed tissue than green soybeans. In comparison, black soybeans contain the most protein (Shurtleff and Aoyagi, 2009). Black and dark brown soybeans are commonly used in Asian culture for both human and animal consumption. These components of edamame breeding line “R07-589” could provide a unique breeding and marketing opportunity not only for its moderate resistance to *M. incognita*, Race 3, but also its unique color and high nutrition content.

Edamame continues to grow in production and popularity in the state of Arkansas. Our studies document the presence of parasitic nematodes and their potential threat to the relatively new industry and that these nematodes are a pest of concern on edamame. Our study also indicates that if the nematode pressure is high enough, nematodes could have a significant impact not only on yield but also seed composition such as protein and moisture levels of edamame, which, in turn, could reduce the marketability of the crop. Where *Heterodera glycines* or *Meloidogyne incognita* populations are present in grower fields, best management strategies should be implemented to avoid potential loss of both edamame yield and quality. Edamame breeding lines were identified that may harbor resistance to *Meloidogyne incognita* and can be utilized to introduce resistant traits into new commercial cultivars. Planting edamame cultivars resistant to plant-parasitic nematodes that are present and in high densities could be a promising management strategy.
